# Justice—Injustice; the Ether Controversy

**Published:** 1880-01

**Authors:** 


### ARTICLE II.

### *Justice—Injustice—the Ether Controversy.*

BY PROF. HODGKIN.

A writer in a late number of *Johnston's Dental Miscellany* takes the remarkable position that the question of priority in the discovery of anæsthesia is unnecessarily revived, and that its merits were finally settled by an act of the Connecticut Legislature in 1847; and that any attempted reversal of that decision in the light of further evidence is ill-timed and possibly illegal. At least such is the inference one may draw from the remarks of this writer, who seems to think that because the new and complete evidence on the subject, which incontestably awards to Dr. Crawford W. Long the honor of being the first to use ether as an anæsthetic in surgery, comes from the State of Georgia, and is announced in a public way by a distinguished Georgia statesman, that therefore such evidence should be ignored. Although why such evidence should be slightly esteemed, he fails to inform any one.

Certainly because the Legislature of Connecticut in 1847 had never heard of Dr. Long or his discovery is no reason why his claims did not exist, nor is it any reason why they are not rightful. There has been an unfairness and disingenuousness about all this matter utterly unworthy of liberal professions such as medicine and dentistry, into whose relations certainly sectionalism should never enter; and to reject the claims of Dr. Long or any one else on such grounds, in the face of such ample evidence, is simply dishonest.

The strictly substantial evidence on this subject—evidence which it is simply fatuous to ignore—is that Dr. C. W. Long on the thirtieth day of March, 1842, gave to young Venable ether, produced anæsthesia, and excised a tumor in his neck; that he repeated the administration *with success* again in June and July of the same year, and also in succeeding years. There are numbers of people now living, physicians, prominent citizens of Jefferson, Jackson County, Georgia, ready to testify, and who do testify to these facts; people who could have no possible interest in the controversy, save in the cause of truth and justice.

The writer of the article in question claims for Dr. Wells, priority of suggestion in the matter of the use of nitrous oxide gas. The official facts are that Wells in 1840, after attempting the administration of gas, said that he "believed a man could be made so drunk on the gas as to have teeth extracted painlessly." But this idea was forty years old at that time, though Wells was doubtless unaware of it, as Sir Humphrey Davy had made this suggestion in the year 1800. It was not until 1844 that Wells first produced anæsthesia with nitrous oxide, *two years after* Long had publicly made known his discovery of the effects of ether.

Indeed the discovery itself was accidental, and was made not by Dr. Long, but by a young gentleman, afterwards his student, who in a frolic gave ether to a little negro boy, produced anæsthesia, and supposed the result to be fatal.

There has been an unjustness and unfairness in this controversy, painful to witness, among men whose only desire should be to know the truth and award the credit where it belongs.

We republish from the *New York Medical Record* for September 6th, one of the best summaries we have seen of the honors done in a public way to the memory of the discoverer of anæsthesia, meantime tending thanks to the family of Dr. Long for placing at our disposal a mass of most interesting documents bearing on the case, with full accounts of the public ceremonies, and other papers.

It is a singular circumstance that of the four men thus concerned in this controversy—Morton, Wells, Jackson and Long—that the first three should have died insane, and the latter of softening of the brain. And of Jackson the unworthy record is that although he was aware of Long's claims, and fully posted by personal interviews and examinations of his priority, he failed to give him any public credit for his discovery.

We have just seen a very beautiful and touching letter from the daughter of Dr. Long, written to Dr. J. J. Caldwell, of Baltimore, acknowledging in grateful terms his endeavors to obtain from the medical profession public credit for the claims of her father, and of his advocacy of the cause of truth and justice in this matter.

